# Comparison between in vitro and in vivo cartilage overloading studies based on a systematic literature review

**DOI:** 10.1002/jor.23910

**Published:** 2018-04-26

**Authors:** Mieke Nickien, Ashley Heuijerjans, Keita Ito, Corrinus C. van Donkelaar

**Affiliations:** ^1^ Department of Biomedical Engineering, Orthopaedic Biomechanics Eindhoven University of Technology P.O. Box 513, 5600MB Eindhoven The Netherlands

**Keywords:** cartilage, mechanics, In vitro, In vivo, post‐traumatic OA

## Abstract

Methodological differences between in vitro and in vivo studies on cartilage overloading complicate the comparison of outcomes. The rationale of the current review was to (i) identify consistencies and inconsistencies between in vitro and in vivo studies on mechanically‐induced structural damage in articular cartilage, such that variables worth interesting to further explore using either one of these approaches can be identified; and (ii) suggest how the methodologies of both approaches may be adjusted to facilitate easier comparison and therewith stimulate translation of results between in vivo and in vitro studies. This study is anticipated to enhance our understanding of the development of osteoarthritis, and to reduce the number of in vivo studies. Generally, results of in vitro and in vivo studies are not contradicting. Both show subchondral bone damage and intact cartilage above a threshold value of impact energy. At lower loading rates, excessive loads may cause cartilage fissuring, decreased cell viability, collagen network de‐structuring, decreased GAG content, an overall damage increase over time, and low ability to recover. This encourages further improvement of in vitro systems, to replace, reduce, and/or refine in vivo studies. However, differences in experimental set up and analyses complicate comparison of results. Ways to bridge the gap include (i) bringing in vitro set‐ups closer to in vivo, for example, by aligning loading protocols and overlapping experimental timeframes; (ii) synchronizing analytical methods; and (iii) using computational models to translate conclusions from in vitro results to the in vivo environment and vice versa. © 2018 The Authors. *Journal of Orthopaedic Research*® Published by Wiley Periodicals, Inc. on behalf of the Orthopaedic Research Society. J Orthop Res 36:2076–2086, 2018.

In articular cartilage, the zonally differentiated collagen fibril structure and embedded hydrophilic proteoglycans (PGs) provide the tissue with its remarkable mechanical properties.^1^, ^2^, ^3^ A decreased structural integrity during osteoarthritis (OA) is associated with an impaired load‐bearing functionality.^4^, ^5^ These mechanical changes also occur within the micro‐environment of the chondrocytes, which can affect their protein production and lead to additional structural changes.^6^ The structure‐mechanics of articular cartilage are thus considered to play a crucial role in OA induction as well as progression.

Although inflammation can also play a major role in the onset of OA,^7^ biomechanical risk factors, such as body weight, joint alignment, and knee trauma, are well established.^8^, ^9^, ^10^ A widely used experimental model for the investigation of OA is therefore to induce cartilage damage by mechanical overloading. The ultimate goal of such research is to determine mechanical thresholds that would induce damage in a specific way to particular parts of the cartilage structure. Examples may be the shear stress or impact energy that would cause damage to the cartilage matrix, or the tensile strain or strain rates that would rupture or de‐structure the collagen fiber network. Understanding such thresholds for healthy and compromised cartilage provides an upper limit to the mechanical perturbations that cartilage can withstand. These may ultimately be taken into account for making decisions on treatment strategies or to advise on post‐operative recovery.

In vitro overloading studies are conducted to assess damage initiation and/or short‐term explant effects providing results within a timeframe of seconds to several weeks, whereas in vivo experiments normally last months up to a year. A major advantage of an in vitro study is the possibility to apply a specific loading protocol in a highly controlled fashion. Once a culture protocol has been established and bioreactors are validated, performing additional in vitro studies is generally cheaper and faster than in vivo studies, and there is no discomfort for test subjects or need for ethical approval. In addition, in vitro the biochemical conditions are controlled and typically kept constant, allowing for comparison between samples based solely on difference of loading. The main advantage of an in vivo study is that it provides a natural biomechanical and biological environment to study OA as a total joint disease. In vivo studies also include inflammation, bone adaptation, and other longer term processes. In vitro studies are thus used to answer more fundamental research questions, while in vivo studies can be used to investigate a response under natural conditions over longer time.

Unfortunately, methodological differences make it challenging to directly compare results between in vitro and in vivo studies. By taking the results from many in vivo and in vitro studies together and categorizing them by outcome parameters, we aim to identify consistent factors in the relationship between mechanical overloading and articular cartilage damage development. Inconsistencies between in vitro and in vivo studies may also be revealed. These may identify in vitro approaches that are not sufficiently representative for in vivo conditions, or they address aspects that can only be studied appropriately in vivo.

The aim of the current review therefore is to (i) identify consistencies and inconsistencies between in vitro and in vivo studies on mechanically‐induced structural damage in articular cartilage, such that variables that would be interesting to further explore using either one of these approaches can be identified; and (ii) suggest how the methodologies of both approaches may be adjusted to facilitate easier comparison and therewith stimulate translation of results between in vivo and in vitro studies. This is anticipated to enhance our understanding of the development of osteoarthritis, and to help replacing part of our in vivo studies by in vitro approaches.

## APPROACH

The PubMed database was searched for relevant papers using various forms and synonyms of the following terms in their title: Cartilage, damage, stress, strain, overload, load, impact, meniscectomy, transection, tear. This yielded 433 papers, from which only those relevant to this review were selected. Papers on in vitro cartilage synthesis (tissue engineering) and/or computational cartilage models were, in general, beyond the scope of this review and therefore largely excluded. However, the role of the latter in connecting in vitro to in vivo is discussed in chapter 5. In vivo studies did not include human clinical studies, because in those studies OA is not experimentally induced. Only publications written in English were considered. The reference lists of selected papers were also searched for additional relevant publications. The search was updated just before submission to the journal of orthopaedic research.

**Figure 1:**
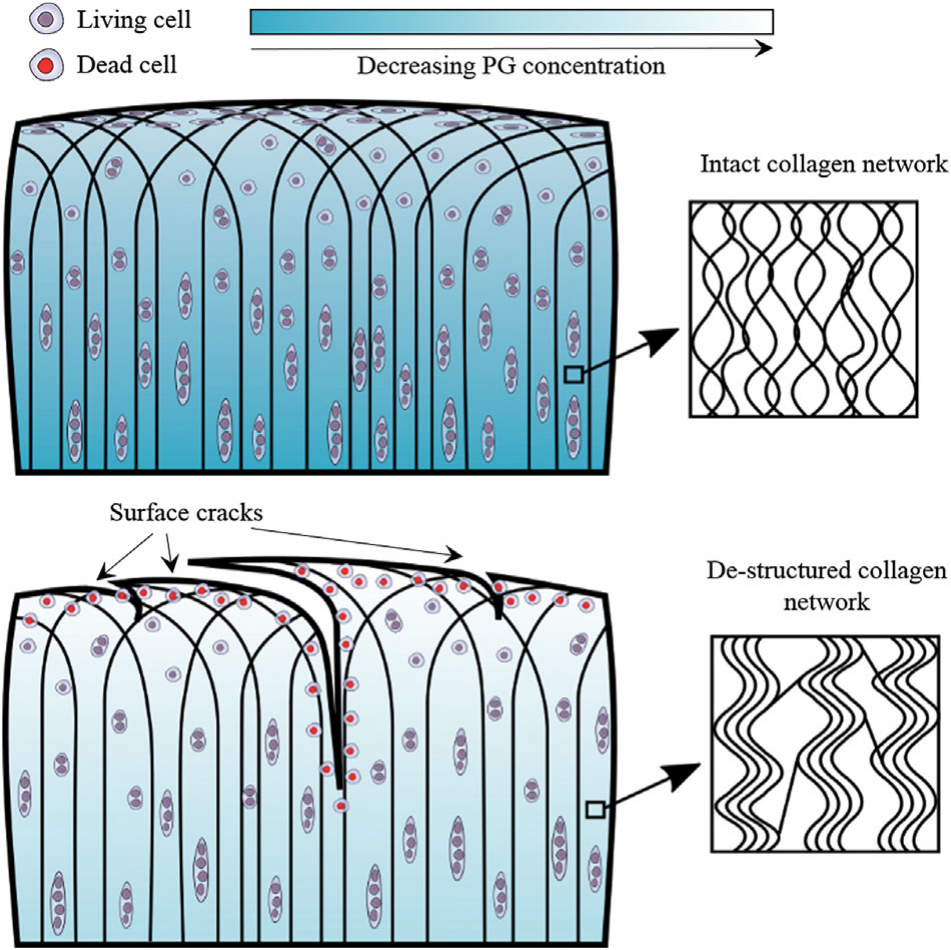
Illustration of possible structural features in the intact (top) and excessively loaded (bottom) cartilage. A higher intensity of blue colour indicates a (locally) higher PG concentration. Viable cells are shown in purple while dead cells are shown in red. In the full‐thickness cartilage constructs the black lines indicate primary fibrillar direction. The isolated boxes show the collagen network on the ultrastructural level.

Papers were classified as reporting on either in vitro or in vivo overloading studies. Information on macrostructural cartilage damage, swelling behavior, chondrocyte viability, collagen network structure, proteoglycans, and tissue mechanics were documented and used to create a map of events following trauma (see Fig. 1 for an illustrative summary of the structural features that were reviewed). The reader is directed to alternative review papers for additional information on genetics, epigenetics, and the expression of smaller proteins which might also affect structure‐mechanics during cartilage degeneration.

## IN VITRO STUDIES; SHORT‐TERM RESULTS

Mechanical overloading in vitro can lead to immediate crack formation in the cartilage layer.^11^, ^12^ *En face* examination of cracked samples reveals that the rupture orientation is initially in the split line direction and that additional fissures are also oriented in this direction or at a constant angle to it.^13^, ^14^, ^15^, ^16^, ^17^, ^18^ Fissures proceed to increasing depths into the cartilage matrix following the arcade‐like architecture of the collagen network, that is, originally parallel to the articular surface and then transitioning into the radial orientation, until finally the fissures run along the calcified boundary and cartilage delamination occurs.^19^, ^20^, ^21^ The probability and severity of surface fissuring following trauma generally increases with higher impact energy,^22^, ^23^, ^24^ applied stress,^19^, ^25^, ^26^, ^27^, ^28^, ^29^, ^30^, ^31^, ^32^, ^33^, ^34^, ^35^ stress rate,^14^, ^26^, ^28^, ^29^, ^30^, ^36^, ^37^ frequency of dynamic loading,^17^ loading duration,^18^, ^28^ and a period of prolonged creep prior to overloading^21^, ^38^. From a certain impact threshold, in this case an impact energy of 0.25 J imposed on 5 mm diameter cartilage explants, it has even been observed that there was damage to the subchondral bone without macroscopic damage to the cartilage.^24^, ^39^ Increasing levels of macrostructural damage however can also be attributed to specimen‐related characteristics, such as a higher degree of degeneration,^20^, ^40^ higher stiffness,^13^ lower level of maturity,^20^ increased in‐plane surface strain,^15^ or decreased thickness of subchondral bone^16^, ^24^. In contrast, cartilage is less prone to fracture when the most superficial layer is removed,^13^, ^19^ or when a pre‐strain of 10% or higher is applied prior to overloading.^41^ The amount and depth of surface fissures in cartilage decreases its surface strain‐limiting abilities, induces tissue swelling,^42^ and alters the compressive load distribution,^43^ which may lead to further mechano‐biological damage.^44^, ^45^

Decreased cell viability following mechanical overloading indicates excessive local strain on a microstructural level.^46^ Both the amount of necrotic and apoptotic cells can increase after injurious loading^47^, ^48^ in any overloaded area in the cartilage.^27^, ^37^, ^49^, ^50^, ^51^, ^52^, ^53^, ^54^ Also, fissures caused by mechanical overloading are always surrounded by dead cells.^25^, ^31^, ^55^, ^56^, ^57^, ^58^, ^59^ Following traumatic compression, the depth of non‐viable cells from the articular surface increases with increasing contact stress,^25^, ^27^, ^49^, ^50^, ^51^, ^60^, ^61^, ^62^ increasing loading duration^48^, ^50^, ^54^, ^60^ (up to a number of cycles^27^), lower maturity level,^51^, ^63^ absence of the superficial layer,^52^ higher impact energy,^24^, ^31^, ^58^, ^64^, ^65^ the amount of cartilage preloading,^38^ and absence of subchondral bone.^16^ The viability response to varying strain rates is rather complex because cartilage behaves in a relatively stiff and incompressible manner under high strain rates, and is more compliant under slower or sustained loading when the water is given time to be expelled.^2^, ^39^, ^62^, ^66^, ^67^ At lower compressive strain rates, chondrocyte death might be found throughout the entire cartilage depth, whereas at higher strain rates viability is only decreased in the superficial layer.^14^, ^29^, ^36^, ^56^, ^62^ This indicates that the most superficial layer experiences higher stresses than the underlying tissue at higher strain rates.^68^ If the strain rates are then further increased the superficial layer of dead cells becomes thicker.^29^, ^67^ Cell viability in the superficial layer seems to be unaffected by the frequency of cyclic intermittent loading.^69^ Cell viability generally keeps decreasing over time when chondral explants are maintained in culture following a traumatic event^30^, ^49^, ^52^, ^64^, ^70^ and this time‐dependent response is dependent on the loading protocol that is used.^48^, ^56^, ^57^, ^63^ During extended culture times cell death also increases in control samples, which complicates the investigation of cell death as a result of trauma.^65^, ^71^ A decreased cell viability leads to an even lower ability of tissue remodeling following trauma^72^ and the release of biochemical factors by perturbed, apoptotic, or necrotic cells.

Changes in bulk collagen content are not usually observed following trauma,^73^, ^74^, ^75^ however resulting structural changes alone can have a large effect on cartilage mechanics. These changes to the collagen network can be due to fibril denaturation^73^, ^74^ or fibril rupture as observed during, for example, surface fissuring, but can also be due to a reduced level of inter‐fibrillar connections.^76^ Such a reduced interconnectivity has been termed network de‐structuring, which is when the network is transformed from its normal highly interconnected “pseudo‐random” appearance^77^ to a less interconnected structure with an overall increased aligned fibrosity.^78^ Associated with this de‐structuring is an increased matrix tendency to soften and swell due to the decreased constraint of proteoglycan swelling.^28^, ^34^, ^37^, ^53^, ^56^, ^64^, ^73^, ^79^, ^80^, ^81^, ^82^ However, the extent of softening following injurious loading may be a combined effect of network de‐structuring and proteoglycan alterations described in the following paragraph. It has further been shown that tissue softening due to excessive loading can precede collagen denaturation,^75^ which seems logical since the interconnections between the collagen fibrils are weaker in tension than the fibrils themselves.^83^ Cartilage exhibits increased swelling and softening with higher loads,^24^, ^53^, ^65^, ^67^, ^75^ reveals more microcracks within the collagen network with higher impact energy or applied stress or stress rate,^84^ and levels of degraded collagen coincide with or come at a later stage than cell death indicating that this is also caused by excessive microstrains.^27^, ^50^, ^61^ The increased compliance within the tissue as a result of collagen network de‐structuring has the potential to affect chondrocyte metabolism and the microstructural response to compression.

In principle, PG release following overloading can be caused by de‐structuring of the collagen network, fracturing of the PGs themselves and/or excessive pressure and diminished boundaries. The reduced constraint from a loosened collagen network on the PGs can also lead to a decreased PG density.^44^ An increased glycosaminoglycan (GAG) release^25^, ^53^, ^73^ and synthesis^28^ and decreased content^11^, ^67^ in overloaded samples compared to controls are normally observed from certain threshold loads.^32^, ^34^, ^36^, ^85^ However, GAG synthesis tends to decrease at higher loads^34^, ^37^, ^85^ as cell viability decreases.^28^, ^29^, ^48^ Changes in GAG content have also been shown to be zonally dependent. It has been hypothesized that GAG loss starts in the transition zone, and is mainly synthesized in the deep zone as a response.^50^ Higher contact stress and prolonged loading lead to increased GAG release and synthesis, and decreased GAG content.^50^, ^64^, ^65^ At similar strains, cyclic compression results in increased PG release when compared to static compression, and the lost PGs are then also smaller in size.^86^ Varying the frequency and duration of intermittent cyclic loading affects PG synthesis and release in a non‐linear and irregular manner.^69^ Furthermore, an increased GAG release has been shown to be associated with cartilage fracture following injury.^59^ Depending on the mechanical protocol used, GAG release can be higher in injured samples compared to controls at varying time‐points following loading.^14^, ^29^, ^30^, ^34^, ^64^, ^71^, ^86^ Because of the simultaneous GAG release and synthesis, it may be possible that no changes in GAG content are observed in some culture studies.^74^ A lower GAG concentration following injury reduces the hydrostatic pressure within cartilage, with the ability to affect both mechanical and metabolic properties.

**Figure 2:**
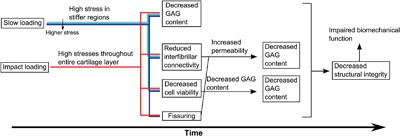
Schematic showing the varying cartilage short‐term responses to overloading in vitro. The immediate occurrences following slow or impact loading are represented by blue or red arrows, respectively. Events occurring at higher local stresses are indicated with a darker shade of blue, as opposed to events occurring at lower local stresses which are indicated with a lighter shade of blue.

In summary, in vitro overloading studies have shown that structural changes following trauma are highly dependent on the mechanical loading applied and the tissue used for experimentation. The resulting structural damage depends on the local stresses induced by the trauma. A reduced level of fibrillar interconnectivity is probably the first sign of excessive internal stress, since tissue weakening can be observed prior to viability changes.^73^ A further increased stress then leads to cell death, collagen denaturation, and eventually surface fissuring. The amount of GAG loss depends on the degree of collagen network de‐structuring and GAG synthesis. Damage on macro‐, micro‐, and nano‐structural level has the potential to alter the local mechanical environment and thus lead to further mechano–biological changes. The overall cartilage response to overloading in vitro has been schematically summarized in Figure 2.

## IN VIVO STUDIES; LONG‐TERM RESULTS

The aim of many in vivo studies on cartilage overloading is to induce post‐traumatic OA using some form of mechanical insult to study OA progression. Such an experimental model can be used to test the effects of a novel treatment strategy following injury or to study individual components in relation to OA progression within a natural environment. Cartilage degeneration can be induced either in a direct fashion (impact or other overloading protocol) or indirectly, for instance by altering joint kinematics.

A way of investigating the effect of mechanical overloading on cartilage in vivo is by introducing a one‐off trauma, such as an impact load, after which the test animal is allowed natural weight bearing without additional damage to any surrounding soft tissue. In a number of those studies, the overloading protocol caused immediate fissuring of the cartilage surface,^87^, ^88^, ^89^, ^90^, ^91^, ^92^ which was reported to be accompanied by cell^87^ and GAG^88^ loss in the uppermost layers. Excessive loading of the whole joint has shown that this superficial fissuring and GAG loss was visible in the tibia but not in the femur.^91^ The rate of damage increase over time following a one‐off trauma varies with animal model and applied trauma. However, there is a general trend that cartilage deteriorates further and does not recover after a trauma causing surface fissuring.^87^, ^88^, ^89^, ^90^, ^92^ The tissue may gradually exhibit chondrocyte clusters, empty lacunae, and increasing GAG loss, and at some point GAG staining may only be visible in the direct vicinity of the cells indicating GAG production but not containment.^88^ In terms of mechanical properties, an immediate reduction in cartilage thickness is associated with a decrease in stiffness, with both thickness and stiffness decreasing further over time.^89^, ^90^ A high impact which immediately damages the subchondral bone but not the cartilage can still result in cartilage degeneration over time, indicating either a delayed direct effect from the impact or a translated effect from the underlying bone.^93^

Cartilage damage can also be induced in vivo by altering the animal model's joint kinematics, for example, by ACL transection, which induces joint instability, or meniscectomy, resulting in directly increased tibia‐femoral contact stresses,^94^ or repeated long‐term overloading by muscle stimulation.^95^ ACL transection, meniscectomy, and repeated long‐term overloading have been shown to induce cartilage degeneration over time in terms of surface fissuring, hypocellularity, loss of structural integrity, GAG increase followed by decrease, and overall tissue softening.^72^, ^87^, ^94^, ^95^, ^96^, ^97^, ^98^, ^99^, ^100^, ^101^, ^102^, ^103^, ^104^, ^105^, ^106^, ^107^, ^108^, ^109^, ^110^, ^111^, ^112^, ^113^, ^114^, ^115^, ^116^, ^117^, ^118^ In these models, the tibia is generally more severely or equally damaged compared to the femur,^110^, ^113^, ^114^, ^118^ and less degeneration has been observed in the patella compared to the femur,^109^ indicating a greater effect on areas that receive the most stress during locomotion. Fissuring and GAG loss tend to worsen over time,^101^, ^102^, ^107^, ^113^ although occasionally the degenerative grade remains relatively stable between varying observation points.^98^, ^103^ The rate of cartilage degeneration also varies with injury modality, that is, when the magnitude and duration of abnormal loading are higher there is an increased rate.^87^, ^101^, ^113^ Similarly, combining various damage modalities such as ACL transection, meniscectomy, and/or application of an impact increases the rate of cartilage degeneration.^96^, ^107^, ^113^

**Figure 3:**
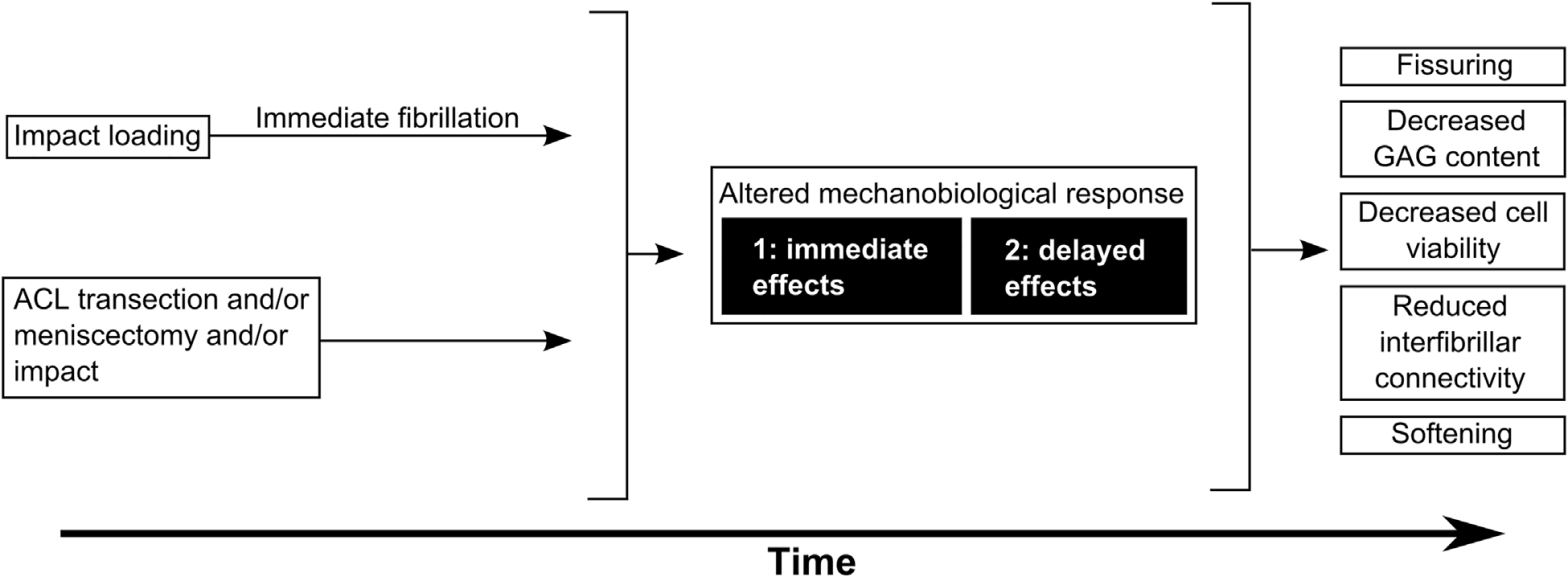
Schematic showing the similar cartilage long‐term response to various in vivo overloading methods. The immediate and delayed effects following trauma are symbolized by black box # 1 and 2, respectively.

Thus, in vivo overloading studies, whether impact‐induced or induced by permanent alterations to joint kinematics, all seem to consistently lead to increasing joint degeneration over time (see Fig. 3). This was expected since many in vivo studies develop or use overloading models which lead to general OA related effects. The damage further seems to be more severe with higher stresses. In the absence of intervention, these studies have not shown that spontaneous repair can counteract the drastic overloading protocols.

## COMPARISON BETWEEN IN VITRO AND IN VIVO OVERLOADING STUDIES

A wide variety of experimental and analytical protocols was employed within the reviewed studies. An example of an experimental difference is the use of various indenter shapes to induce cartilage damage. The effects of such differences could not be elucidated from this review and would be best explored in a direct comparative experimental study. The current review can be interpreted as “multiple overloading pathways leading to degeneration,” bearing in mind that these pathways may have similarities but are not necessarily the same. An overall challenge that remains is to identify which factors in these pathways play important roles, in vitro and/or in vivo.

In general, the results of in vitro and in vivo studies do not contradict each other. It has been shown with both study types that after an extremely high impact the subchondral bone is damaged, while the cartilage initially stays intact. At less high impacts, immediate cartilage fissuring, decreased cell viability, collagen network de‐structuring, an overall decreased GAG content, and an overall damage increase over time are reported for both in vitro and in vivo. Both study types further reveal the low ability of cartilage to recover from cell death or structural damage to the collagen network as a result of overloading. Tissue viability and a functional collagen organization may therefore be important targets for novel therapeutic treatments of early cartilage degeneration. That the outcomes of in vitro and in vivo studies do not contradict each other, encourages further development and extension of in vitro systems to study in vivo effects, which may reduce and refine the use of animals. It would also be interesting to investigate whether specific parameters (e.g., stress or strain rate), which are shown to affect cartilage in vitro, can be controlled in vivo to prevent damage due to overloading. These rationales justify efforts to further align in vitro and in vivo study methodologies.

One way to increase resemblance between in vitro and in vivo experimental set‐ups is by attempting to make in vitro loading protocols more physiological. To assess whether this is feasible one should consider the available experimental set‐ups (see Table 1). Each of the four set‐ups shown in Table 1 has its advantages and disadvantages making it suitable for specific research targets. Most of the in vitro experiments performed until today were explant studies, using either living (i.e., cultured) or dead tissue. Although these explant systems allow for an accurately defined loading protocol, the relatively small samples with their unconstrained boundaries likely affect tissue response and may therefore not be representative of loading within a total joint. Systems that are currently perhaps underexplored and could potentially bridge the gap between explant and in vivo studies are total joint motion simulators. The loading that can be applied with such systems is more physiological than loading regimes commonly used in explant studies, while it allows for more controlled loading than is possible in vivo. A major drawback of these systems is that it is still very challenging to accurately determine the exact loading within a joint. However, the joint motion simulator may for instance be employed to develop short‐term post‐operative recommendations for in vivo joint movement.

**Table 1:** Overview of Experimental Methods to Assess the Cartilage Response to Overloading

	Whole joint **↓**	Explant **↓**
	Uncertainties about distribution of stresses in the joint	Unphysiological boundary conditions Possible to use human cadaveric tissue
–		
Living tissue →	Animal (in vivo) studies	Culture studies
Includes repair Long‐term	Real‐life response Low motion control Uncertainties about translation to human Need to get ethical approval	Can investigate cell phenotype Simulated environment
–		
Dead tissue →	Total joint movement simulator	Structure‐mechanical testing
No repair Short‐term Possible to use human cadaveric tissue	Motion control Possible to use human cadaveric tissue	Highest controllability and repeatability Cheapest

An alternative method to align loading protocols of in vitro and in vivo studies is to restrict motion of subjects in an in vivo study to investigate the effect of altered loading. It has been shown that interventions such as joint distraction,^119^, ^120^, ^121^, ^122^, ^123^ high tibial osteotomy,^124^ and bracing lead to decreased cartilage degeneration and improved patient‐reported outcome. However, it is complicated to determine the isolated effect of loading since it is impossible to measure in vivo loads. For bracing, it has been shown that improvement in pain is small‐to‐moderate while improvement in gait mechanics was moderate‐to‐high.^125^, ^126^ It has also been demonstrated that the placebo effect can play a role in patient experience.^127^ Thus, the exact contribution of load‐reduction in motion‐alternating treatments has not yet been elucidated and further research with an objective approach needs to be performed.

**Figure 4:**
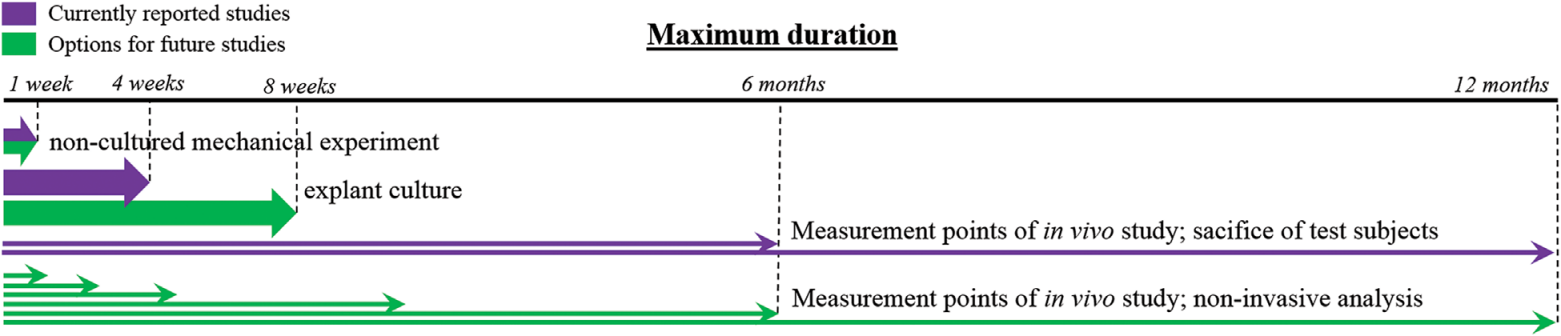
Timeline of previous overloading studies and proposed timeline for future overloading studies.

Another important difference between current in vitro and in vivo methods is the analytical timepoints. The maximum duration of explant incubation reported in the papers included in this review was 4 weeks. The number of reviewed papers on in vivo studies showing results of 4 weeks or less is small (7 out of 32). In vivo studies generally lasted several months up to a year for the larger species (lapine, murine, ovine, and primates). The number of measurement timepoints is usually limited to 2 or 3, because of ethical considerations: It is often required to sacrifice test subjects for each timepoint. However, with the recent advances on in vivo cartilage imaging and corresponding image analysis^128^ it will be possible in the future to add more timepoints for analysis of the same subject. This will help decreasing the number of subjects needed and increasing the accuracy of time‐dependent measurements (see Fig. 4). Simultaneously, ex vivo incubation systems are vastly improving, as it has been shown that cartilage‐on‐bone explants can stay intact for up to 8 weeks.^129^ These explants can also be compressed and biochemically supplemented as required, and are thus increasingly resembling the in vivo environment.^130^ Such systems may in time provide an opportunity to omit short‐term animal studies (see overlap between explant incubation time and in vivo studies in Fig. 4). Thus, increased overlap in timeframes of in vitro and in vivo studies may help elucidate the isolated effect of loading, particularly in the in vivo set‐up where the effect of loading cannot be isolated, and it may help comparing results between in vivo and in vitro.

An additional option to further align in vitro and in vivo results is by making the analytical methods more comparable. The papers reviewed here showed a wide variety of reported results, both of structural and mechanical aspects. Also some papers, particularly those on in vivo studies, merely report Mankin score as an outcome, which is a widely accepted and validated cartilage damage score but does not specifiy the nature of this damage. This also complicates comparison with in vitro studies. Related to the lack of information of structural damage of these studies, the effect of individual factors observed following trauma, such as surface cracking, network de‐structuring, or cell death, are currently underexplored in vivo. Increased reporting of such information would make in vitro and in vivo studies more comparable, thereby potentially increasing our understanding of how load affects degeneration.

The final way in which in vitro studies can be linked to in vivo studies is through computational modeling. A full review of the current state of available computational models is beyond the scope of this review, but it is acknowledged that these models can provide valuable support. Computer models, once thoroughly validated, may assist in translation from geometrically simple to complex conditions, from short to long‐term effects, or across length‐scales. First, geometrical complexity generally challenges the accuracy of the mechanical or biological behavior that can be incorporated. For example, some recently developed models for studying patient‐specific knee joint structure and personal gait mechanics to predict the tissue's degenerative behaviour^131^, ^132^, ^133^ are geometrically complex, and may be useful to compare to in vivo data. However, such models can only be computed with less sophisticated cartilage material models that cannot be related to damage mechanisms at the tissue scale. In contrast, advanced material models^134^ are too computationally intensive to be used in patient‐specific whole joint models, but they are helpful when interpreting mechanical details of in vitro studies on explant materials. Second, translating from short‐term (related to in vitro studies) to long‐term effects (related to in vivo studies) requires algorithms for damage progression related to repeated mechanical overloading.^132^, ^135^ In addition, incorporation of biological or pathological effects such as inflammatory conditions are important. Capturing such effects in a quantitative manner, with mathematical equations, is challenging, yet important to make future computational models more versatile and applicable to predict long‐term changes. Simulations of bone adaptation,^136^ and fracture healing (study comparing various theories by Isaksson et al.^137^), demonstrate the feasibility of making long‐term predictions of tissue changes. However, making such long‐term predictions for cartilage is still in its infancy. Third, translations across length‐scales (multi‐scale or multi‐level approaches; reviewed by Halloran at al.^131^), allow connections between the level of the musculoskeletal system, the joint, the tissue or the cell. The larger two levels represent the level at which in vivo experiments are performed, whereas the smaller scales relate more to in vitro studies. Finally, another aspect unique to computer modeling is the ability to compute parameters that are difficult to assess experimentally. This may contribute to translating results from in vivo to in vitro studies and vice versa, as distributions of the same variable can be computed, and observed effects can be related to this distribution. It should be recognized that the validity of computational models generally depends on implementation accuracy, the level of validation, and the suitability of the model to address the pertaining question. The choice of the model to use depends on the particular research question, and care must be taken not to over‐interpret results in the domain in which the model is weak. Yet, the advantage of modeling is that they can be tuned either way, and therefore form an intermediate between in vitro and the in vivo results.

## CONCLUSIONS

In conclusion, comparison of in vitro and in vivo studies based on study outcome parameters is complicated by the underlying experimental differences. However, the outcomes of in vitro and in vivo studies do not contradict each other. This encourages further improvement of in vitro systems, where loading can be the only experimental variable, to study effects in the highly complex complete joint in vivo. Efforts to bridge the gap between in vitro and in vivo studies could include (i) bringing in vitro set‐ups closer to in vivo or vice versa, for example, by modifying loading protocols and/or experimental timeframes; (ii) synchronizing the analytical methods of both study types; and (iii) using computational models as a tool to corroborate in vitro results against in vivo predictions. Although one aspiration is to create in vitro models that closely resemble the in vivo situation, it will be highly challenging, if at all possible, to simulate the complete complex in vivo environment over time as depicted by black box #2 in Figure 3. For the foreseeable future it will therefore, for ethical reasons, be unavoidable to perform animal testing prior to application in humans. Appropriate in vitro tests prior to animal studies however may reduce the amount of animal studies and should therefore be further developed.

## AUTHORS’ CONTRIBUTIONS

Substantial contributions to research design, or the acquisition, analysis or interpretation of data by MN and AH. Drafting the paper or revising it critically by MN, AH, KI, and CCvD. Approval of the submitted and final versions: MN, AH, KI, and CCvD.
